# β-Elemene alleviates cisplatin resistance in oral squamous cell carcinoma cell via inhibiting JAK2/STAT3 pathway in vitro and in vivo

**DOI:** 10.1186/s12935-022-02650-7

**Published:** 2022-07-31

**Authors:** Haiye Wang, Yingyi Ma

**Affiliations:** 1 Special Dental Care Clinic, the First Affiliated Hospital of Zhengzhou University, Henan Stomatological Hospital, Zhengzhou, 450000 Henan China; 2 The First Affiliated Hospital of Zhengzhou University, Henan Stomatological Hospital, No.1 Jianshe East Road, Erqi District, Zhengzhou, 450000 Henan China

**Keywords:** β-Elemene, Cisplatin, Apoptosis, JAK2, STAT3 signaling pathway, Oral squamous cell carcinoma

## Abstract

**Objective:**

To investigate the effect of β-Elemene (β-Ele) on the cisplatin sensitivity of OSCC cells and its mechanism in vitro and in vivo.

**Methods:**

The human OSCC cell lines Tca-8113 and the cisplatin-resistant cell line Tca-8113-CDDP were cultured with β-Ele or/and cisplatin. The cytotoxicity of cisplatin or β-Ele, cell viability, cell cycles and apoptosis were detected. And the expression of JAK2/STAT3 related protein were detected. The xenograft tumor model of OSCC was established in nude mice and treated with cisplatin and/or β-Ele. The volume and weight of the transplanted tumor was measured, and the expression of p-JAK2 and p-STAT3 and cell apoptosis in the xenograft tumor tissues were detected.

**Results:**

The combination of β-Ele and cisplatin significantly suppressed the cell proliferation, induced cell cycle arrest, promoted the apoptosis of Tca-8113-CDDP cells, and suppressed the activation of JAK2/STAT3 signaling pathway. The rescue experiments suggested that β-Ele enhanced cisplatin sensitivity via down-regulating JAK2/STAT3 signaling pathway. In vivo, β-Ele and cisplatin synergistically suppressed the tumor growth and induced apoptosis, and down-regulated the expression of p-JAK2 and p-STAT3.

**Conclusions:**

β-Ele inhibits the cell viability and enhances the cisplatin sensitivity of OSCC by blocking the activation of JAK/STAT3 signaling pathway in vitro and in vivo, and the combination of β-Ele and cisplatin maybe a novel treatment for OSCC.

## Introduction

Oral squamous cell carcinoma (OSCC) is the most common malignancy in the oral cavity. There were approximately 53,000 new cases of OSCC were reported in 2019 [1]. Surgery, drug-based chemotherapy or combination therapy are the main treatments of OSCC in clinical. However, the overall survival rate of OSCC remains around 50% [2, 3]. *Cis*-dichlorodiammine-platinum (CDDP), as a chemotherapy drug, is widely used in the treatment of OSCC. Nevertheless, most OSCC patients are resistance to cisplatin in the clinical practice, which may be the major reason of their low survival rate [4]. Therefore, enhancing the sensitivity to cisplatin of OSCC cells is of great significance for improving the prognosis and survival of OSCC patients.

β-Elemene (1-methyl-1-vinyl-2,4-diisopropenyl-cyclohexane, β-Ele), extracted from Chinese herb *Curcumae Rhizoma,* is a bioactive compound with a broad-spectrum anti-tumor effect [5]. The previous studies have reported that β-Ele can inhibit cell proliferation, induce cell cycle arrest and enhance cisplatin-induced cell death in human solid tumors, such as bladder cancer, lung cancer through regulation of extracellular signal regulated kinase (ERK) and phosphoinositol 3-kinase (PI3K)/Akt signaling pathways [6, 7]. And the β-Ele also can reduce the dose of other chemotherapy drugs and adverse effect, which may be a promising adjuvant treatment drug [8]. But, the mechanism underlying its anti-tumor effect in OSCC and its effect on the sensitive of OSCC cells to cisplatin treatment has not been reported. The aim of this study was to explore the effect of β-Ele on resistant to cisplatin in OSCC and elucidate the underlying molecular mechanism.

## Material and methods

### Cell culture

Human OSCC cell lines SCC-9 (CBP60428), CAL-27 (CBP60427) and Tca-8113 (CBP60426) were purchased from the Cell Bank of Chinese Academy of Sciences (Shanghai, China). All cells were cultured in Dulbecco’s Modified Eagle Medium (DMEM, Gibco, USA) supplemented with 10% fetal bovine serum (FBS, Gibco, USA) and 1% penicillin/streptomycin at 37 ℃ with 5% CO_2_. And the cells were sub-cultured when 90% confluent.

According to the previously described methods [9], cisplatin-resistant cell line Tca-8113-CDDP, was derived from cisplatin-sensitive cell lines Tca-8113 by treating with gradually increasing doses of cisplatin (Sigma, USA) in cell culture medium until the survival cells exhibited a normal growth pattern.

### MTT assays

The cisplatin or β-Ele cytotoxicity and the cell viability were detected by MTT (3-(4,5-dimethylthiazol-2-yl)-2,5-diphenyltetrazolium bromide) assay. For detection of cisplatin and β-Ele cytotoxicity, cells were seeded in 96-well plates with a density of 5 × 10^3^ cells/well. Then, Tca-8113 and Tca-8113-CDDP cells were treated with a various concentration of cisplatin (0, 0.5, 1, 2, 4, 8, 16, 32 μg/ml) or β-Ele (Yuanda Pharmaceutical Co. Dalian, China) in gradient concentrations (0, 20, 40, 60, 80, 100 μg/ml). After cultured for 48 h, the cells were incubated with 20 µl MTT (Promega, USA, 5 mg/ml) for 4 h. After MTT removed, 200 μl DMSO was added. The optical density (OD) at 570 nm was determined using microplate reader (Bio-Rad, USA). The cell viability were calculated according to the OD values, as follows: cell viability = (OD_drug _− OD_blank_)/(OD_0drug _− OD_blank_). The half-maximal inhibitory concentration (IC_50_) of cisplatin or β-Ele was determined from the dose-responses curves.

As for the cell proliferation, cells seeded in 96-well plates at 5 × 10^3^ cells/well were cultured for 24 h, 48 h or 72 h, then examined by MTT assay as above. And the proliferation curve was plotted with time point as the X-axis and OD value as the Y-axis.

### Flow cytometry assays

For cell cycle analysis, the cells were seeded into 6-well plates at 5 × 10^4^ cells/well, and cultured for 24 h at 37 ℃ with 5% CO_2_. Then the cells were treated with drugs for 48 h, washed with cold PBS for twice and fixed with 70% ethanol for 24 h at 4 ℃. After washed with PBS, the cells were incubated with RNase A (0.1 mg/ml), and stained with propidium iodide (PI, Sigma, USA) for 30 min in the dark according to the manufacturer’s protocol. The cell cycles were analyzed by FACSan flow cytometry (BD Biosciences, USA).

For cell apoptosis analysis, the cells were seeded into 6-well plates at density of 5 × 10^4^ cells/well for 24 h. After treatment with drugs for 48 h, the cells were collected and washed with cold PBS, subsequently stained with Annexin V-APC and 7-AAD according to the protocol of Annexin V-APC/7-AAD Apoptosis Detection Kits (Multi Sciences Biotech, Hangzhou, China). Then the cell apoptosis rates were assessed by FACSan flow cytometry (BD Biosciences, USA).

### Real-time fluorescence quantitative PCR assays (RT-qPCR)

Total RNA from cells was isolated with TRIzol reagent (Thermo Fisher, USA), and was reverse transcribed into cDNA using a PrimeScript^™^ RT reagent Kit (Takara, Japan). RT-qPCR detection was conducted on 7900HT Fast Real-Time PCR System (Applied Biosystems, USA) with SYBR Green qPCR Mix. The relative expression was calculated using 2^−∆∆Ct^ method, with β-actin as internal reference.

### Western blot analysis

The total protein was extracted by RIPA buffer (Sigma, USA) with protease inhibitor. The proteins were separated by sodium dodecyl sulfate polyacrylamide gel electrophoresis (SDS-PAGE) and transferred to polyvinylidene fluoride (PVDF) membranes. After blocked in 5% nonfat milk for 1 h, the membranes were incubated with appropriate concentration of primary antibodies, including cleaved caspase-3, Bax, Bcl-2, STAT3, p-STAT3^Y705^, JAK2, phospahorlated (p)-JAK2^Y1007/1008^, GAPDH (Abcam, UK) overnight at 4 ℃. Then the horseradish peroxidase (HRP)-conjugated secondary antibody were incubated at room temperature for 2 h. The protein bands were detected by enhanced chemiluminescence according to the manufacturer’s instructions and analyzed using Image-Pro Plus software (National Institutes of Health, USA) with β-actin as internal reference.

### Nude mice xenograft tumor model

Forty BALB/c nude mice (4–6 weeks) were purchased from SLAC Experimental Animal Center (Shanghai, China) and housed under specific pathogen free conditions with a 12 h dark/light cycle at (25 ± 2) ℃. Tca-8113-CDDP cells (5 × 10^6^) were subcutaneously injected into the right axilla of mice. Then the mice were randomly divided into four groups (10 mice in each group): control group, β-Ele group, CDDP group, β-Ele+CDDP group, and received intraperitoneal administration of PBS, β-Ele (45 mg/kg), and/or cisplatin (4 mg/kg) every three days (all drug were added PBS and make to 0.1 ml), respectively, from the average tumor size reached 0.1 cm^3^. The dose was according to the reported dosage application [10, 11]. The width and length of tumors were measured once every three days with a caliper, to calculate the tumor size [Volume (mm^3^) = width^2^ × length/2]. The mice were sacrificed by intraperitoneal injection of pentobarbital sodium (50 mg/kg) at day 27 after tumor cell inoculation. The xenograft tumors were obtained and weighted. Half of the tissues were fixed in 10% formalin and embedded in paraffin, serial Sections. (5 μm), the other tissues were stored in liquid nitrogen. All animal experiments were approved by the Animal Research Committee of the First Affiliated Hospital of Zhengzhou University.

### Immunohistochemistry (IHC)

The tumor tissue sections were taken for immunohistochemistry to determine the expression of p-JAK2 and p-STAT3. After dewaxing and hydration, the antigens were retrieved and endogenous peroxidase was blocked by 3% H_2_O_2_ for 20 min. After blocking with 5% BSA, the sections were incubated with primary antibodies: p-JAK2, p-STAT3 (Abcam, UK) overnight at 4 ℃. Then the secondary antibodies were incubated for 1 h at 37 ℃. DAB substrate was used and the sections were counterstained by hematoxylin. The positive expression of brown-yellow granules were observed under a inverted microscope (Olympus Corporation, 200× , 400×). Immunohistochemistry in tissues was quantified using Image ProPlus v.7.0. H-score was based on the staining intensity and percentages of stained cell. Four score categories in immunostaining intensity: negative (0), weak (1), moderate (2), and strongly (3). H-score (range of 0–300) was calculated as follow: H-score = [(1 × % weakly stained cell) + (2 × %moderately stained cells)+(3 × % strongly stained cells)] [12]. Five visual field were randomly selected to calculate the averaged H-score for each section.

### TUNEL assay

Apoptosis rate in the xenograft tumor was measured by using TUNEL assay kit-Cy3 (Beyotime, Shanghai, China) according to the protocol of manufacture. Nuclei were counterstained with DAPI reagent (Life technology, USA). Positive cells were visualized using fluorescence microscope (Olympus, Japan).

### Statistical analysis

All the data were analyzed by SPSS21.0 (SPSS Inc., Chicago, USA) and graphs were drawn with GraphPad Prism Version 6.1 (GraphPad Software, Inc., USA). The data were presented as ($\overline{x} \pm s$). Student’s t test was used to analyze the significance of difference between the two group. *P* < 0.05 were considered significance.

## Results

### β-Ele suppressed the cell viability of OSCC cells

**Fig.1:**
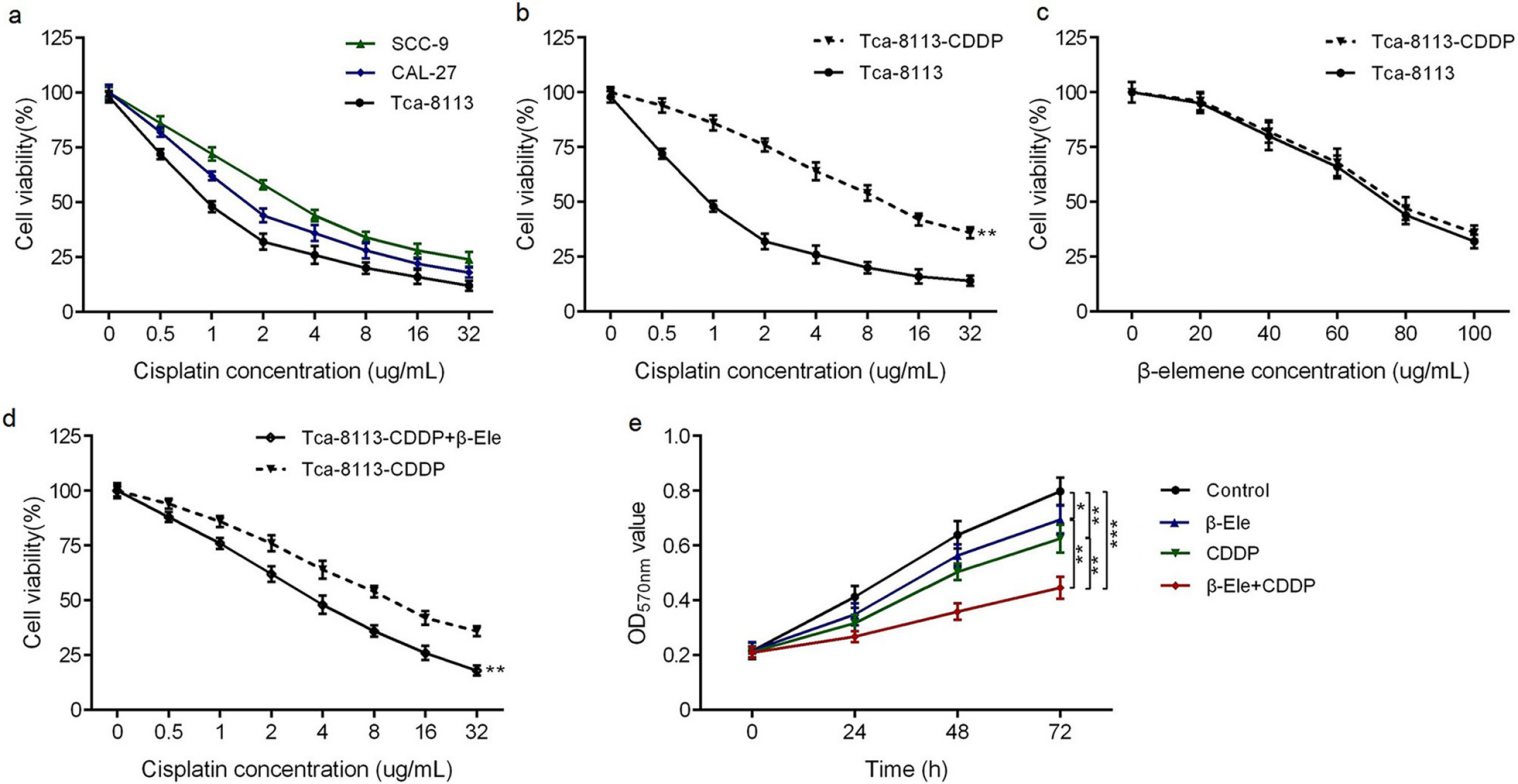
IC_50_ values of cisplatin or β-Ele in OSCC cells and the enhancement of β-Ele on cisplatin sensitivity in Tca-8113-CDDP cells. **a** The cell viability of Tca-8113, CAL-27 and SCC-9 cells treated with different concentration of cisplatin (0–32 μg/ml) for 48 h; **b** the cell viability of Tca-8113 and Tca-8113-CDDP cells treated with different concentration of cisplatin (0–32 μg/ml) for 48 h; **c** the cell viability of Tca-8113 and Tca-8113-CDDP cells treated with different concentration of β-Ele (0–100 μg/ml) for 48 h; **d** the cell viability of Tca-8113-CDDP cells treated with different concentration of cisplatin (0–32 μg/ml) in the absence or presence of β-Ele (40 μg/ml) for 48 h; **e** the proliferation curve of Tca-8113-CDDP cells treated with β-Ele (40 μg/ml) or/and cisplatin (3.5 μg/ml); *P < 0.05, **P < 0.01, ***P < 0.001. *CDDP* cisplatin, *β-Ele* β-Elemene, *OD* optical density

MTT assay was performed to confirm the cisplatin sensitivity of OSCC cells (Tca-8113, CAL-27 and SCC-9 cells) after cultured for 48 h, the results showed that cisplatin inhibited the cell viability in a dose-dependent manner (Fig. 1a). The IC_50_ value of cisplatin in Tca-8113 cell was 0.97 μg/ml, significantly lower than that in CAL-27 (1.88 μg/ml) and SCC-9 cells (3.54 μg/ml), respectively. So Tca-8113 cell was selected as the object to construct drug-resistant cells. The IC_50_ of cisplatin in Tca-8113-CDDP cell was 9.70 μg/ml, significantly higher than that of Tca-8113 cells (Fig. 1b), indicating successful induction of cisplatin resistant cells.

Then, MTT assay was performed to assess the anti-proliferative effect of β-Ele on OSCC cells. Tca-8113 and Tca-8113-CDDP cells were incubated with β-Ele at different concentrations (0, 20, 40, 60, 80, 100 μg/ml) for 48 h. The results showed that β-Ele suppressed the growth and proliferation of Tca-8113 and Tca-8113-CDDP cells in dose-dependent manners (Fig. 1c). There was no significant difference on the IC_50_ values of β-Ele in Tca-8113 cells (74.42 μg/ml) and Tca-8113-CDDP cells (76.32 μg/ml), indicating that β-Ele had a similar anti-proliferative activity toward both in cisplatin-sensitive and cisplatin-resistant OSCC cells lines in vitro.

### β-Ele potentiated the cisplatin chemosensitivity in cisplatin-resistant OSCC cells

To further explore the enhancing effect of β-Ele on cisplatin chemosensitivity in cisplatin-resistant OSCC cells, the Tca-8113-CDDP cells were incubated with cisplatin at different concentration (0, 0.5, 1, 2, 4, 8, 16, 32 μg/ml) in the absence or presence of β-Ele at 40 μg/ml, a non-cytotoxic concentration which exert minimal effect on cell growth. It was found that the IC_50_ value of cisplatin for Tca-8113-CDDP cells treated with β-Ele were changed to 3.53 μg/ml, significantly lower than that without β-Ele (9.70 μg/ml) (Fig. 1d). The data suggested that β-Ele significantly enhanced the chemosensitivity to cisplatin in Tca-8113-CDDP cells.

Based on the results of concentration screening, Tca-8113-CDDP cells were treated with the β-Ele (40 μg/ml) in combination with cisplatin [3.5 μg/ml, IC_50_ value of cisplatin in Tca-8113-CDDP cells in the present of β-Ele (40 μg/ml)]. Then the cell proliferation of Tca-8113-CDDP cells with β-Ele or cisplatin, or the combination of β-Ele and cisplatin were detected by MTT (Fig. 1e). It was found that the combination of β-Ele and cisplatin significantly inhibited the proliferation of Tca-8113-CDDP cells, compared to Tca-8113-CDDP cells treated with β-Ele or cisplatin alone, revealing that the combination of β-Ele and cisplatin imposed a more powerful inhibiting effect on the proliferation in cisplatin-resistant OSCC cells.

### β-Ele promoted cisplatin-induced cell cycle arrest and apoptosis in cisplatin-resistant OSCC cells

**Fig.2:**
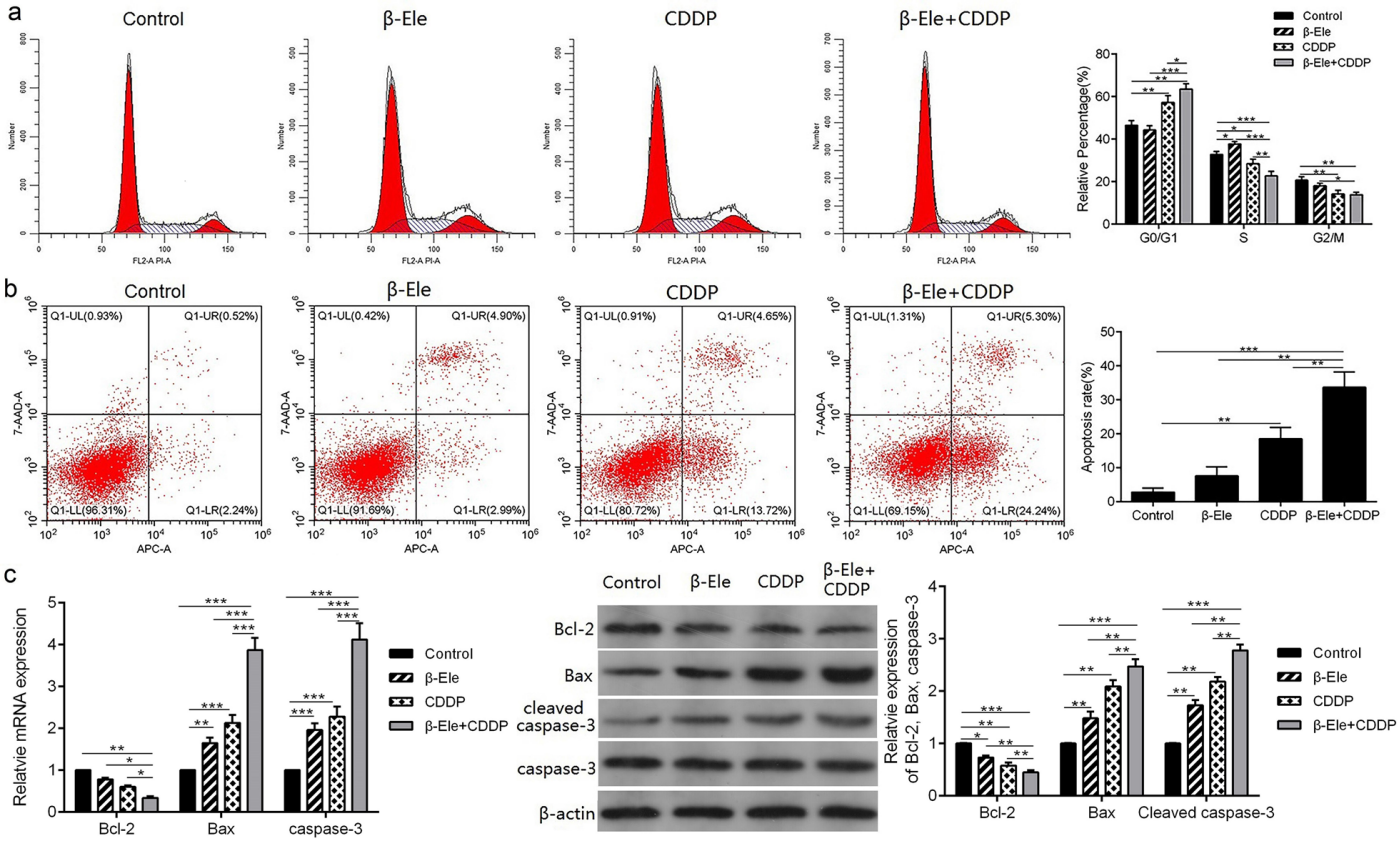
β-Ele promoted cisplatin-induced cell cycle arrest and apoptosis in Tca-8113-CDDP cells. **a** The cell cycle of Tca-8113-CDDP cells treated with β-Ele or/and cisplatin detected by flow cytometry assays; **b** the apoptosis rate of Tca-8113-CDDP cells treated with β-Ele or/and cisplatin detected by flow cytometry assays; **c** the mRNA and protein expression of anti-apoptotic protein Bcl-2, pro-apoptotic protein Bax and caspase-3 in Tca-8113-CDDP cells treated with β-Ele or/and cisplatin detected by RT-qPCR and Western blot. *P < 0.05, **P < 0.01, ***P < 0.001. *CDDP* cisplatin, *β-Ele* β-Elemene

It is well known that β-Ele and cisplatin can induce cell cycle arrest, inhibit cell proliferation, So the cell cycles were detected in Tca-8113-CDDP cells treated with β-Ele or cisplatin alone and combination of β-Ele and cisplatin for 48 h (Fig. 2a). The results showed that, treatment with β-Ele induced S phase arrest and cisplatin induced G_0_/G_1_ phase arrest in Tca-8113-CDDP cells. While the combined treatment of β-Ele and cisplatin significantly reduced the cell percentage in S phase, increased the cell percentage in G_0_/G_1_ phase, suggesting the combination of β-Ele and cisplatin induced Tca-8113-CDDP cells arrest at G_0_/G_1_ phase.

Furthermore, the apoptosis was evaluated in Tca-8113-CDDP cells treated with β-Ele or cisplatin alone and combination of β-Ele and cisplatin for 48 h (Fig. 2b). The results revealed that compared with the Tca-8113-CDDP cells treated with β-Ele or cisplatin alone, the combined treatment of β-Ele and cisplatin obviously increased the percentage of apoptosis cells. Consistently, the expression of cleaved caspase-3 and pro-apoptotic protein Bax increased both in mRNA and protein, while the expression of anti-apoptotic Bcl-2 was reduced in the cells co-treated with β-Ele and cisplatin (Fig. 2c). These results indicated that β-Ele combined with cisplatin induced cell cycle arrest and enhanced the cell apoptosis in cisplatin-resistant OSCC cells.

### β-Ele enhanced cisplatin sensitivity via down-regulating JAK/STAT3 signaling pathway

**Fig.3:**
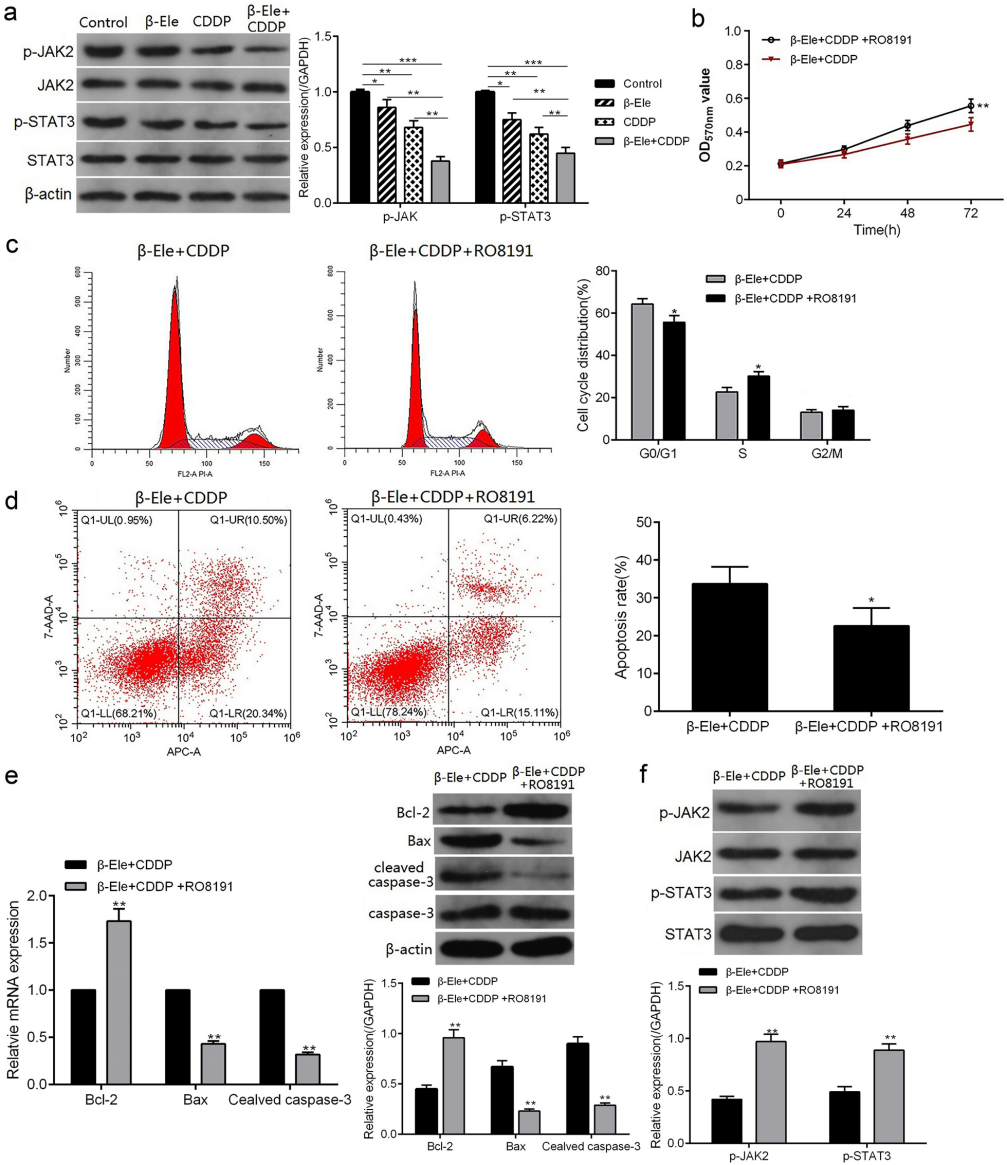
β-Ele enhanced cisplatin sensitivity via down-regulating JAK2/STAT3 signaling pathway. **a** The expression of p-JAK2 and p-STAT3 detected by western blot in Tca-8113-CDDP cells treated with β-Ele or/and cisplatin; **b** the proliferation of Tca-8113-CDDP cells treated with β-Ele + cisplatin, and JAK activator RO8191 detected by MTT assays. **c** The cell cycles of Tca-8113-CDDP cells treated with β-Ele + cisplatin, and JAK activator RO8191 detected flow cytometry assays; **d** the apoptosis rate of Tca-8113-CDDP cells treated with β-Ele + cisplatin, and JAK activator RO8191 detected flow cytometry assays. **e** The mRNA and protein expression of Bcl-2, Bax, and caspase-3 in the Tca-8113-CDDP cells treated with β-Ele + cisplatin, and JAK activator RO8191 detected by RT-qPCR and Western blot. **f** The expression of p-JAK2 and p-STAT3 in the Tca-8113-CDDP cells treated with β-Ele + cisplatin, and JAK activator RO8191 detected by Western blot.*P < 0.05, **P < 0.01, ***P < 0.001. *CDDP* cisplatin, *β-Ele* β-Elemene, *OD* optical density

Emerging evidences indicated that aberrant activation of the JAK/STAT3 signaling might be involved in OSCC development and progression [13]. The results of Western blot analysis showed that treated with β-Ele or cisplatin alone, the combination of β-Ele and cisplatin all markedly inhibited the expression of phosphorylated JAK2 (p-JAK2) and p-STAT3 in Tca-8113-CDDP cells, and the combination of β-Ele and cisplatin were the lowest (Fig. 3a).

In order to understand whether modulation of the JAK/STAT3 pathway was involved in β-Ele promoting sensitivity to cisplatin in cisplatin-resistant OSCC cells, the cell proliferation, cell cycle, apoptosis and the expression of JAK2 and STAT3 in the Tca-8113-CDDP cells treated with the combination of β-Ele and cisplatin, and JAK activator RO8191 were detected by the MTT assay, flow cytometry assay and Western blot analysis. As shown in Fig. 3b–e, RO8191 could promoted the proliferation, promoted cell cycle transition from G0/G1 phase to S phase and inhibited the apoptosis of Tca-8113-CDDP cells treated with the combination of β-Ele and cisplatin. And the expression of p-JAK2 and p-STAT3 were elevated in the Tca-8113-CDDP cells co-treated with β-Ele, cisplatin and RO8191 (Fig. 3f). These data demonstrated that β-Ele enhanced cisplatin sensitivity of cisplatin-resistant OSCC cells via inhibiting JAK/STAT3 signaling pathway.

### β-Ele suppressed the growth of Tca-8113-CDDP xenograft in nude mice

**Fig.4:**
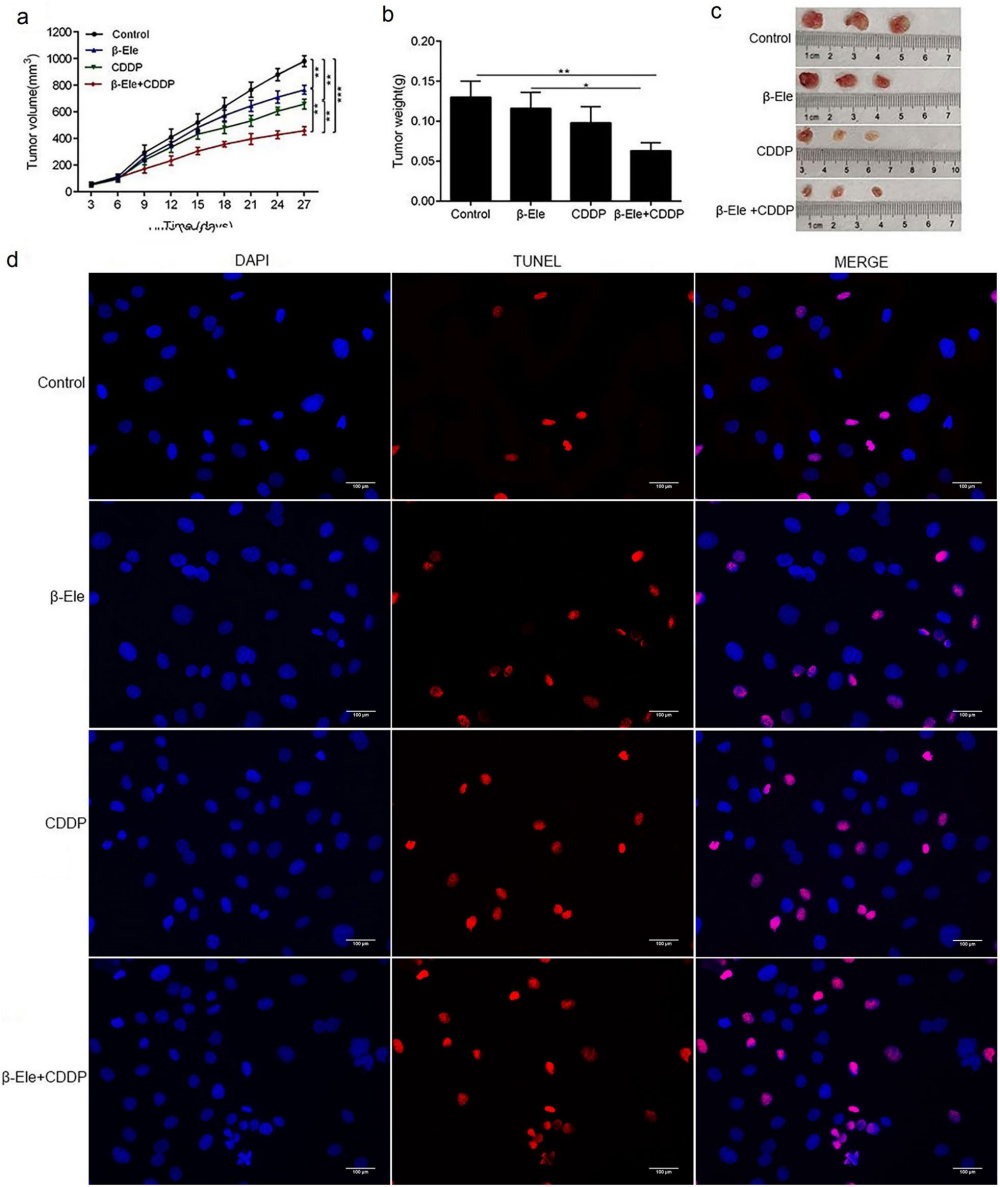
β-Ele and cisplatin synergistically suppressed the growth of xenograft tumor and induced apoptosis in nude mice. **a** Tumor volumes were measured once every 3 days from day 0 to day 27 in each group of mice. **b** Tumor weight was measured at day 27 following treatment in each group of mice. **c** The xenograft tumors derived from Tca-8113-CDDP cells, after mice sacrificed at day 27. **d** TUNEL staining was used to detect the apoptosis in the tumor tissues in each group of mice, Scale bar = 100 μm. *P < 0.05, **P < 0.01, ***P < 0.001. *CDDP* cisplatin

**Fig.5:**
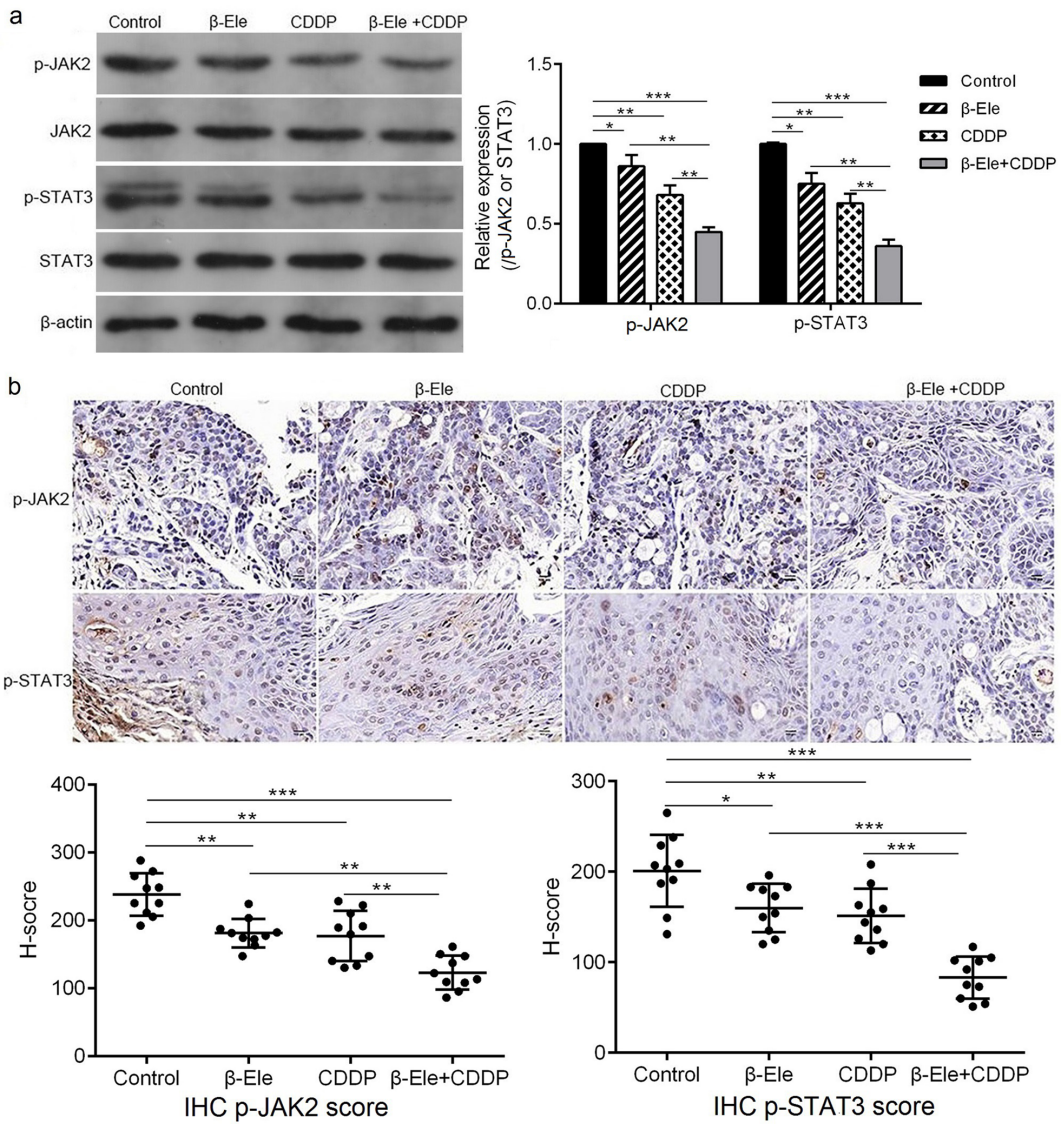
β-Ele and cisplatin synergistically inhibit the JAK2/STAT3 signal pathway. **a** The expression of p-JAK2 and p-STAT3 in the tumor tissues in each group of mice detected by Western blot. **b** The expression of p-JAK2 and p-STAT3 were examined and quantified by immunohistochemistry, Scale bar = 100 μm. *P < 0.05, **P < 0.01, ***P < 0.001. *CDDP* cisplatin, *IHC* immunohistochemistry

Tca-8113-CDDP were used to construct an OSCC xenograft model in nude mice to explore the anti-cancer effect of the combination of β-Ele and cisplatin in vivo. As shown in Fig. 4a–c, compared with the control, β-Ele and cisplatin groups, both of tumor volume and tumor weight in the β-Ele + CDDP group were decreased significantly. The results of TUNEL assay (Fig. 4d) showed that β-Ele combined with cisplatin promoted cell apoptosis in the tumor tissues. The Western blot analysis (Fig. 5a) of tumor tissues showed that the β-Ele combined with cisplatin markedly suppressed the expression of p-JAK2 and p-STAT3. Similarly, the down-regulation of p-JAK2 and p-STAT3 in the tumor tissues of the mice co-treated with β-Ele and cisplatin were confirmed by IHC assay and H-score (Fig. 5b). All these results supported that β-Ele combined with cisplatin effectively inhibited the tumor growth and induced apoptosis maybe by inhibiting JAK/STAT3 signaling pathway in vivo.

## Discussion

As a highly effective chemotherapy drug, cisplatin is considered as a mainstay of combination chemotherapy for patients with OSCC, which is significant to improve the survival rate [14]. However, the effectiveness of cisplatin is limited by the drug resistance and toxicity side effects, particularly during prolonged or high dose chemotherapy [15, 16]. Recently, β-Ele, a traditional Chinese medical herbs with anticancer activity, has been approved for clinical use to treat multiple solid tumors, such as lung cancers [17], esophageal squamous cell carcinoma [18], and hepatocellular carcinoma [19]. In generally, β-Ele can inhibit and kill tumor cells by inhibiting cell proliferation, arresting cell cycle and inducing apoptosis. A study of Cai et al. [20] showed that β-Ele suppressed the viability, invasion and migration of bladder cancers and inducing apoptosis significantly by up-regulating PETN and suppressing Akt phosphorylation (p-Akt). And Liang et al. [21] also found that β-Ele exerted its anti-tumor effects on esophageal cancer by inhibiting proliferation, migration and invasion, inducing apoptosis of esophageal cancer cells. Also, β-Ele has been shown to increase the sensitivity to chemoradiotherapy and chemotherapy of cancer cells, thereby improving the clinical treatment response [22]. In breast cancer, Su et al. [23] revealed that β-Ele and 5-Fu synergistically inhibited the cell viability, proliferation, migration and invasion, promoted apoptosis, and enhanced the effect of 5-FU through multiple mechanisms. Gan et al. [24] suggested that β-Ele inhibited the proliferation and enhanced cisplatin-induced apoptosis of bladder cancer cells in vitro through the ROS-MAPK signaling pathway. In lung cancer, β-Ele adjuvant treatment could overcome the radioresistance of NSCLC and reverse the epithelial mesenchymal transition and cancer stem cells transdifferentiation induced by radiation via the Prx-1/NF-kB/iNOS pathway [25]. Zhou et al. [26] demonstrated that β-Ele could reverse the drug resistance of cisplatin(DDP)-resistance of lung adenocarcinoma cells via promoting Beclin-1-induced autophagy. In ovarian cancer, a previous study demonstrated that β-Ele sensitized the chemoresistant ovarian cancer cells to cisplatin-induced apoptosis and augmented the cytoxicity of cisplatin [27]. A recent study showed that β-Ele combined with paclitaxel inhibited growth and induced apoptosis of ovarian cancer cells, suggesting that β-Ele enhanced the anti-ovarian cancer effects of paclitaxel through regulation of the STAT3/NF-κB signaling pathway [28]. In the present study, the effect of β-Ele on resistant to cisplatin in OSCC was evaluated in vitro and in vivo*.* The results found that β-Ele could suppressed the cell viability of OSCC cells, and β-Ele and cisplatin synergistically suppressed the cell viability, induced cell cycle arrest and promoted apoptosis in Tca-8113-CDDP cells, suggesting β-Ele potentiated the cisplatin chemosensitivity in cisplatin-resistance OSCC cells. Meanwhile, the results in vivo also found that β-Ele enhanced the inhibitory effect of cisplatin on the growth of OSCC xenograft tumors and induced the apoptosis in tumor tissues. It is generally accepted that induction of cell apoptosis was a main goal of chemotherapy for human malignancies and an important mechanism of anti-tumor efficacy of cisplatin [29]. The results of Western blot also showed that the expression of anti-apoptotic protein Bcl-2 was decreased, pro-apoptotic protein Bax was increased, and caspase-3 was activated by the combination of β-Ele and cisplatin both of in vitro and in vivo. It was concluded that β-Ele sensitized cisplatin-induced apoptosis in cisplatin-resistant OSCC cells.

Numerous studies have revealed that β-Ele could modulate multiple molecular pathways such as ERK1/2, PI3K/Akt/mTOR, ROS-MPAK, JAK/STAT3 involved in carcinogenesis [22, 23, 30]. It is reported that JAK/STAT3 signaling pathway plays critical roles in progression of many human solid tumors such as breast cancer [31], ovarian cancer [32] and OSCC [33]. A previous study showed that β-Ele inhibited the growth of nasopharyngeal carcinoma cells via inactivation of STAT3 [34]. And Huang et al. [35] reported that β-Ele enhanced the cisplatin-induced inhibition of proliferation and promotion of apoptosis in gingival squamous cell carcinoma via inhibiting the activation of JAK2/STAT3 signaling pathway. The results in this study also found that β-Ele and cisplatin synergistically inhibited the expression of p-JAK2 and p-STAT3 in vitro and in vivo. And JAK activator RO8191 promoted the proliferation, inhibited the G0/G1 phase arrest and apoptosis, and up-regulated the expression of p-JAK2 and p-STAT3 in Tca-8113-CDDP cells treated with β-Ele + cisplatin, suggesting that β-Ele enhanced cisplatin sensitivity via inhibiting the activation of JAK2/STAT3 signaling pathway in cisplatin-resistant OSCC cells.

## Conclusion

In conclusion, the results from the present study indicated that β-Ele suppressed the cell viability of OSCC cells and promoted the sensitivity of cisplatin by blocking the JAK2/STAT3 signaling pathway in CDDP-OSCC in vitro and in vivo*,* suggesting β-Ele may restore cisplatin sensitivity in cisplatin-resistant OSCC cells. Therefore, the combination of β-Ele and cisplatin may be considered as a promising therapeutic strategy to overcome drug resistant for the treatment of OSCC patients. However, there was still some deficiencies in the study: the effect of β-Ele on the sensitivity towards cisplatin in OSCC cells and its mechanism need to be further verified in other cisplatin resistant cell lines. And whether there was difference on the mechanisms between cisplatin-resistant OSCC cells and their parental counterparts was also need to be explore in the follow-up study.

## Data Availability

The dataset supporting the conclusions of this article is available from the corresponding author.
